# Influence of social deprivation index on in-hospital outcomes of COVID-19

**DOI:** 10.1038/s41598-023-28362-0

**Published:** 2023-01-31

**Authors:** Parag Goyal, Edward Schenck, Yiyuan Wu, Yongkang Zhang, Aayush Visaria, Duncan Orlander, Wenna Xi, Iván Díaz, Dmitry Morozyuk, Mark Weiner, Rainu Kaushal, Samprit Banerjee

**Affiliations:** 1 Department of Medicine, Weill Cornell Medical College, 1320 York Avenue, New York, NY 10021 USA; 2 NewYork-Presbyterian Hospital, 525 East 68th Street, New York, NY 10065 USA; 3 Department of Population Health Sciences, Weill Cornell Medical College, 425 East 61St Street, New York, NY 10065 USA; 4 Center for Pharmacoepidemiology and Treatment Sciences, Rutgers Institute for Health, Health Care Policy, and Aging Research, New Brunswick, NJ USA; 5 Department of Pediatrics, Weill Cornell Medical College, New York, NY USA; 6 New York, USA

**Keywords:** Viral infection, Outcomes research

## Abstract

While it is known that social deprivation index (SDI) plays an important role on risk for acquiring Coronavirus Disease 2019 (COVID-19), the impact of SDI on in-hospital outcomes such as intubation and mortality are less well-characterized. We analyzed electronic health record data of adults hospitalized with confirmed COVID-19 between March 1, 2020 and February 8, 2021 from the INSIGHT Clinical Research Network (CRN). To compute the SDI (exposure variable), we linked clinical data using patient’s residential zip-code with social data at zip-code tabulation area. SDI is a composite of seven socioeconomic characteristics determinants at the zip-code level. For this analysis, we categorized SDI into quintiles. The two outcomes of interest were in-hospital intubation and mortality. For each outcome, we examined logistic regression and random forests to determine incremental value of SDI in predicting outcomes. We studied 30,016 included COVID-19 patients. In a logistic regression model for intubation, a model including demographics, comorbidity, and vitals had an Area under the receiver operating characteristic curve (AUROC) = 0.73 (95% CI 0.70–0.75); the addition of SDI did not improve prediction [AUROC = 0.73 (95% CI 0.71–0.75)]. In a logistic regression model for in-hospital mortality, demographics, comorbidity, and vitals had an AUROC = 0.80 (95% CI 0.79–0.82); the addition of SDI in Model 2 did not improve prediction [AUROC = 0.81 (95% CI 0.79–0.82)]. Random forests revealed similar findings. SDI did not provide incremental improvement in predicting in-hospital intubation or mortality. SDI plays an important role on who acquires COVID-19 and its severity; but once hospitalized, SDI appears less important.

## Introduction

Given the profound impact of the COVID-19 pandemic, research on COVID-19 has become an important priority across the world, especially as it relates to prediction of outcomes^1^. Beyond patient demographics and traditional clinical characteristics, social factors have emerged as important predictors of outcomes. For example, prior work from our group and others have shown that higher social deprivation index (SDI) and deprived living environment are associated with hospitalization for COVID-19^2,3^, with mortality and other poor outcomes both in the US and in the UK.

The SDI is a neighborhood-level marker of social disadvantage related to a dearth of health care resources^4^. SDI is especially important in the United States because individuals from regions with higher SDI (more disadvantaged) have higher risk of disease and often experience limited access to care, resulting in an unmet need in healthcare and poor patient outcomes^4^. High SDI is also associated with worse post-hospitalization outcomes in myriad diseases, and has been postulated to result from limited resources for recovery, limited continuity of care, and a greater burden of comorbidities^5^. While it is well-known that SDI plays an important role on processes prior to a hospitalization and after hospitalization^5^, its impact on in-hospital processes is less well-characterized.

Given emerging evidence regarding the influence of SDI and other social risk indicators on multiple aspects of the COVID-19 pandemic^2,6^, we sought to examine the importance of SDI on the prediction of in-hospital COVID-19 outcomes including intubation and in-hospital mortality. Understanding the influence of SDI on in-hospital outcomes’ prediction could provide important insights on care delivery during a pandemic, and potentially identify an important source of significant disparities for in-hospital outcomes of COVID-19. To address this gap in knowledge, we leveraged one of the largest electronic health record (EHR) datasets of hospitalized patients, derived from three major health systems in New York City (NYC)^7^ one of the first epicenters—during multiple phases of the pandemic (from March 1, 2020 to February 8, 2021).

## Methods

Inclusion criteria were: (1) adults (≥ 18 years of age) (2) confirmed COVID-19 by positive RT-PCR test or ICD-10 diagnosis (3) admission to emergency department (ED) or hospital between March 1, 2020 and February 8, 2021. Patients living in a nursing home prior to their index presentation were excluded as zip codes in EHR may not represent their residence. The resulting cohort included 30,016 unique patients with confirmed COVID-19.

### Exposure: social deprivation index

We linked clinical data using patient’s residential zip-code with social data at zip-code tabulation area (ZCTA) to compute the Social Deprivation Index (SDI)^8^ for 2020 using publicly available sources^9^. SDI is a composite of six socioeconomic characteristics (income, education, employment, housing, household characteristics and transportation) determined at the ZCTA level. We mapped patients’ residential zip codes onto ZCTAs. We categorized all ZCTAs into quintiles based on SDI score.

### Outcomes

The two outcomes of interest were in-hospital intubation and in-hospital mortality. Intubation was defined as mechanical ventilation during hospital stay based on the presence of relevant orders and procedure codes. In-hospital mortality was defined as deaths that occurred during the hospitalization recorded in hospital EHR or reflected in the Diagnosis Related Group.

### Patient characteristics

We examined demographics, baseline comorbidities, and vital signs at admission. Demographics included age, sex, race (White or non-White), and ethnicity (Hispanic or non-Hispanic). Established diagnosis codes^10^ were used to identify baseline comorbidities including hypertension, diabetes, coronary artery disease, heart failure, chronic obstructive pulmonary disease, asthma, cancer, obesity, and hyperlipidemia. Vital signs that were robustly captured by the participating health systems included systolic and diastolic blood pressure, and Body Mass Index (BMI) at admission.

### Statistical analysis

To predict the two binary outcomes (intubation and mortality), we considered two methods—a logistic regression and random forests (RF). First, we constructed a sequence of models with logistic regression to evaluate the incremental effect of each group of predictors. Model 1included demographics, comorbidity, and vital signs; we added SDI quintile to construct Model 2, added time since the start of the pandemic to construct Model 3, and finally added an SDI by time interaction to construct Model 4.

Similar models (except Model 4) were constructed with Random Forests, a machine learning algorithm that automatically models complex interactions between predictors. Model performance was estimated by Area Under the Receiver Operating Characteristic curve (AUROC) using a five-fold cross-validation and its 95% confidence interval reported. Missing data in predictors (range 3.6–12%) were imputed with random forest^11^ to produce an imputed dataset.

### Ethical Information

Weill Cornell IRB (#20-04021948) approved this study and determined that this study meets exemption requirements at HHS 45 CFR 46.104(d). All data management and analysis were conducted in a manner that is HIPAA-compliant.

## Results

### Patient characteristics

Among N = 30,016 COVID-19 patients, the median Inter-quartile range (IQR) age was 59.5 (43.2–72.4) years, 50.8% were males, 63.5% were non-White race and 36.4% had Hispanic ethnicity. The most common comorbid conditions were hypertension (53.6%), hyperlipidemia (38.6%), and diabetes (32.9%) (Table 1). Compared to the group with the lowest SDI (1st quintile), the group with highest SDI (5th quintile) had a higher proportion of non-White race and Hispanic ethnicity, had higher prevalence of each of the comorbid conditions, and presented to the hospital earlier in the pandemic.

**Table 1:** Baseline characteristics of hospitalized Covid-19 patients included in the study across SDI (Social Deprivation Index) quintiles.

	Overall (N = 30,012)	Social Deprivation Index (SDI) quintiles					
		1 (N = 5098)	2 (N = 1082)	3 (N = 1590)	4 (N = 1586)	5 (N = 20,656)	*P-*Value
Demographics							
Race, No. (%)							0.007
White	8490 (30.8)	2035 (46.7)	658 (63.0)	827 (54.7)	664 (44.8)	4306 (22.5)	
Ethnicity, No. (%)							< 0.001
Hispanic	10,920 (41.0)	1899 (45.0)	152 (15.6)	301 (21.7)	316 (23.1)	8252 (44.1)	
Age, No. (%), y							0.038
> 64	12,263 (40.9)	2192 (43.0)	439 (40.6)	652 (41.0)	643 (40.5)	8337 (40.4)	
45–64	9992 (33.3)	1622 (31.8)	372 (34.4)	538 (33.8)	511 (32.2)	6949 (33.6)	
25–44	6692 (22.3)	1114 (21.9)	233 (21.5)	351 (22.1)	388 (24.5)	4606 (22.3)	
18–24	1065 (3.6)	170 (3.3)	38 (3.5)	49 (3.1)	44 (2.8)	764 (3.7)	
Male, No. (%)	15,247 (50.8)	2452 (48.1)	570 (52.7)	863 (54.3)	857 (54.0)	10,505 (50.9)	< 0.001
Time, median (IQR)							
Weeks since March 1, 2020,	9.3 (4.7,41.7)	13.4 (5.1,42.1)	33.7 (5.3,43.1)	10.4 (4.7,42.2)	9.9 (4.9,42.0)	8.6 (4.6,41.3)	
Vital signs at admission (Median (IQR)							
Systolic blood pressure	126 (113,140)	124 (112,139)	126 (114,140)	125 (113,141)	126 (115,140)	126 (113,140)	0.016
Diastolic blood pressure	74 (66,83)	75 (68,83)	77 (69,84)	75 (66,83)	76 (66,84)	74 (65,82)	< 0.001
BMI	28 (25,33)	28 (24,32)	29 (25,33)	27 (24,32)	28 (24,32)	28 (25,33)	< 0.001
In-hospital Outcomes, No. (%)							
Intubation	2902 (9.7)	496 (9.7)	84 (7.8)	130 (8.2)	132 (8.3)	2060 (10.0)	0.007
Mortality	3554 (11.8)	488 (9.6)	88 (8.1)	142 (8.9)	156 (9.8)	2680 (13.0)	< 0.001
Comorbidities, No. (%)							
Cancer	5909 (19.7)	910 (17.9)	209 (19.3)	329 (20.7)	305 (19.2)	4156 (20.1)	0.005
Asthma	4696 (15.6)	564 (11.1)	122 (11.3)	218 (13.7)	188 (11.9)	3604 (17.4)	< 0.001
COPD	4019 (13.4)	381 (7.5)	113 (10.4)	210 (13.2)	214 (13.5)	3101 (15.0)	< 0.001
CAD	7544 (25.1)	859 (16.8)	285 (26.3)	477 (30.0)	440 (27.7)	5483 (26.5)	< 0.001
Heart failure	4198 (14.0)	534 (10.5)	114 (10.5)	197 (12.4)	194 (12.2)	3159 (15.3)	< 0.001
Diabetes	9889 (33.0)	1077 (21.1)	286 (26.4)	425 (26.7)	466 (29.4)	7635 (37.0)	< 0.001
Hyperlipidemia	11,580 (38.6)	1312 (25.7)	425 (39.3)	659 (41.4)	616 (38.8)	8568 (41.5)	< 0.001
Hypertension	16,084 (53.6)	2016 (39.5)	553 (51.1)	819 (51.5)	825 (52.0)	11,871 (57.5)	< 0.001

SDI quintiles: 1(Least Disadvantaged), 2(slightly disadvantaged), 3(moderately disadvantaged), 4(more disadvantaged) and 5(most disadvantaged).

*IQR* interquartile range, *CAD* Coronary Artery Disease, *COPD* Chronic obstructive pulmonary disease.

### Intubation

**Figure 1:**
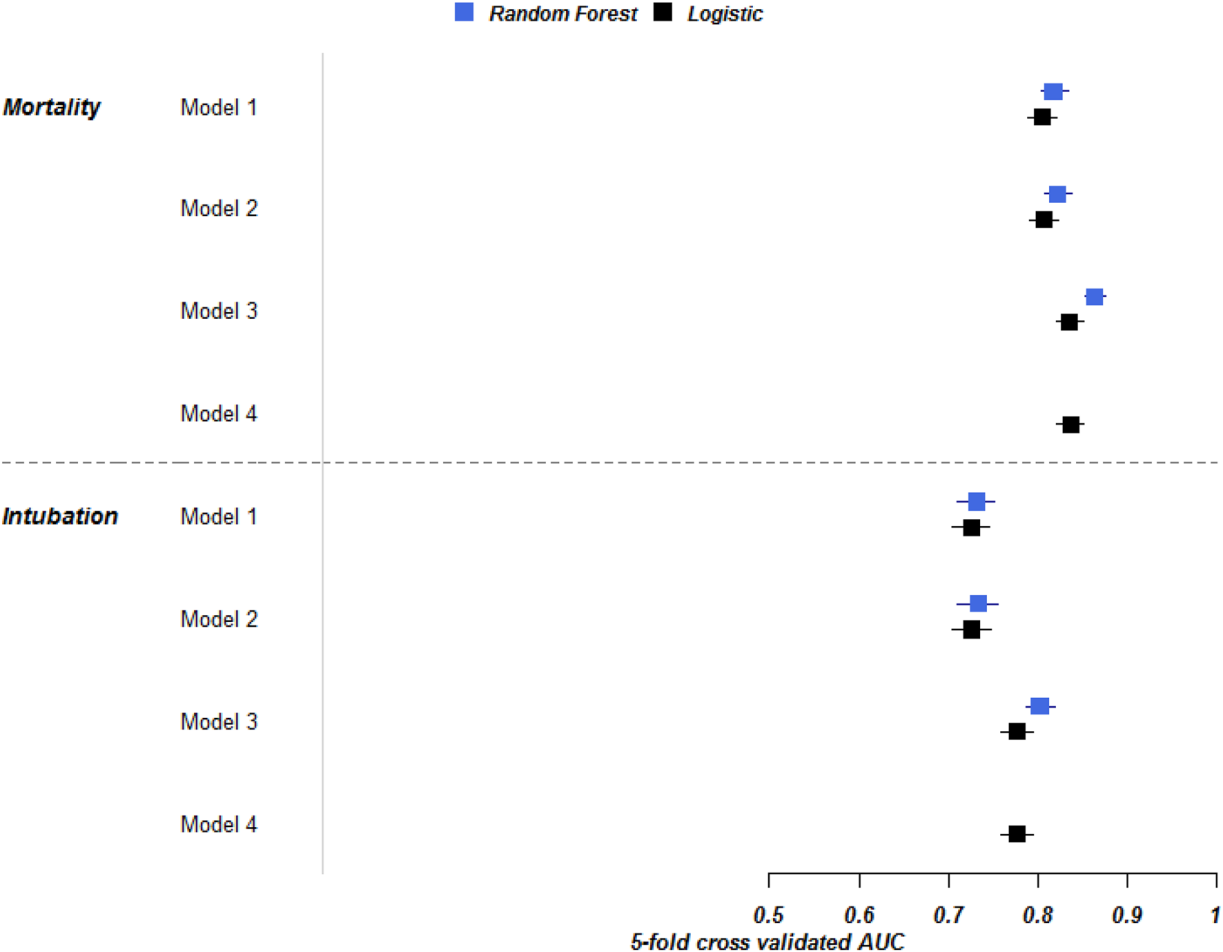
Fivefold cross validated AUROCs of four models using random forest (blue) and logistic regression(black). Model 1 included demographic (gender, race, age, ethnicity), vital signs (BMI, systolic and diastolic blood pressure) and comorbidities (see Table 1); Model 2 added SDI quintiles to Model 1; Model 3 added time (weeks since March 1st, 2020) to Model 2 and Model 4 (logistic regression only) added time x SDI quintile interaction to Model 3.

In a logistic regression model, Model 1 (demographics, comorbidity, and vitals) predicted intubation with moderate accuracy (AUROC = 0.73; 95% CI 0.70–0.75). The addition of SDI in Model 2 did not improve accuracy (AUROC = 0.73; 95% CI 0.71–0.75). The addition of time in Model 3 increased accuracy (AUROC = 0.78; 95% CI 0.76–0.79) compared to Model 2. The addition of an interaction between SDI quintiles and time did not improve prediction (AUROC = 0.78; 95% CI 0.76–0.79). Results from the RF showed similar results (Fig. 1).

### In-hospital mortality

In a logistic regression model, Model 1 (demographics, comorbidity, and vitals) predicted mortality accurately (AUROC = 0.80; 95% CI 0.79–0.82). The addition of SDI in Model 2 did not improve prediction (AUROC = 0.81; 95% CI 0.79–0.82). The addition of time in Model 3 increased accuracy (AUROC = 0.84; 95% CI 0.82–0.85) compared to Model 2. The addition of an interaction between SDI quintiles and time did not improve prediction (AUROC = 0.84; 95% CI 0.82–0.85). Results from the RF showed similar results (Fig. 1).

## Discussion

This study, of over 30,000 patients hospitalized for confirmed COVID-19 in NYC, found that neither SDI nor its interaction with time provided incremental value in predicting in-hospital intubation or death. This suggests that SDI based on a patient’s neighborhood did not influence outcomes once hospitalized for COVID-19 beyond known clinical risk factors, and this did not change over the course of the pandemic.

The importance of social determinants of health has garnered a spotlight in the United States over the past couple years in the setting of national events including COVID-19^12^. Our group previously showed that SDI was associated with hospitalization for COVID-19 and all-cause mortality; but did not consider in-hospital events^2^. This study extends prior findings by indicating that SDI does not predict adverse events beyond demographic and clinical predictors once medical attention is sought.

Our findings are reassuring given concerns about the impact of implicit bias related to social determinants of health on provision of care and associated outcomes during the COVID-19 pandemic^13,14^. Concerns about implicit bias were especially relevant at the peak of the pandemic during which some hospitals had to make plans for rationing care^15,16^. Our findings reveal that SDI did not have a major influence on in-hospital outcomes at any point of the pandemic including at the peak. Given these observations, interventions to address disparities as they relate to SDI should focus on the community rather than the hospital. For example, increased efforts to improve vaccinations rates in high SDI-regions may be especially important to improve outcomes for vulnerable populations. With emerging data about the long-term sequelae of COVID-19^17^, lack of healthcare resources may exacerbate negative impact of “long COVID, suggesting the potential utility of additional resources (e.g. paid sick-leave, housing support etc.) for COVID-19 survivors living in high SDI-regions.”

The strengths of this study are—first, inclusion of several major health systems in NYC, making our findings more generalizable than prior studies based on single institutions; second, the study time-period of 1-year since the beginning of the pandemic, thereby capturing the evolution of clinical knowledge and experience, practice habits, and evidence for therapies over the course of the pandemic; third, use of logistic regression and random forest to model high-level interactions that may not otherwise be easily discerned.

The important limitations are—first, findings are limited to NYC, and may not be generalizable to other regions of the country; second, SDI measures neighborhood level rather than individual level social disadvantage. Consequently, some individual patients may have more or less disadvantage than their neighborhood SDI; third, the complex interplay between SDI, overall health and COVID-19 infection makes risk estimates of predictors biased (collider bias)^18^, therefore we focus on prediction accuracy only; finally, clinical predictors were limited to those robustly captured across all health systems and we did not have data on some known predictors (e.g. respiratory rate) and health behaviors (e.g. diet).

## Conclusion

SDI did not provide incremental improvement in predicting in-hospital intubation or mortality beyond known demographic and clinical predictors. SDI likely plays an important role on who acquires COVID-19, and its severity; but once hospitalized, SDI appears less important. Future interventions to address SDI-related disparities should focus on improving health of the community before acquiring COVID-19, such as through vaccination efforts.

## Data Availability

The datasets analyzed in this study are not publicly available because the data involved de-identified Electronic Health Records of patients served by several academic health centers in New York, NY and housed in a secure data warehouse.
